# Liquid Metal Grid Patterned Thin Film Devices Toward Absorption-Dominant and Strain-Tunable Electromagnetic Interference Shielding

**DOI:** 10.1007/s40820-024-01457-7

**Published:** 2024-07-17

**Authors:** Yuwen Wei, Priyanuj Bhuyan, Suk Jin Kwon, Sihyun Kim, Yejin Bae, Mukesh Singh, Duy Thanh Tran, Minjeong Ha, Kwang-Un Jeong, Xing Ma, Byeongjin Park, Sungjune Park

**Affiliations:** 1 Department of Polymer-Nano Science and Technology, Department of Nano Convergence Engineering, Jeonbuk National University, Jeonju, 54896 Republic of Korea; 2 School of Chemical Engineering, Sungkyunkwan University (SKKU), Suwon, 16419 Republic of Korea; 3 Composites Research Division, Korea Institute of Materials Science, Changwon, 51508 Republic of Korea; 4 School of Materials Science and Engineering, Gwangju Institute of Science and Technology, Gwangju, 61005 Republic of Korea; 5 School of Materials Science and Engineering, and Sauvage Laboratory for Smart Materials, Harbin Institute of Technology (Shenzhen), Shenzhen, Guangdong 518055 People’s Republic of China

**Keywords:** Absorption-dominant electromagnetic interference shielding, Liquid metals, Soft and stretchable electronics, Thin film devices, Tunable electromagnetic interference shielding

## Abstract

The demand of high-performance thin-film-shaped deformable electromagnetic interference (EMI) shielding devices is increasing for the next generation of wearable and miniaturized soft electronics. Although highly reflective conductive materials can effectively shield EMI, they prevent deformation of the devices owing to rigidity and generate secondary electromagnetic pollution simultaneously. Herein, soft and stretchable EMI shielding thin film devices with absorption-dominant EMI shielding behavior is presented. The devices consist of liquid metal (LM) layer and LM grid-patterned layer separated by a thin elastomeric film, fabricated by leveraging superior adhesion of aerosol-deposited LM on elastomer. The devices demonstrate high electromagnetic shielding effectiveness (SE) (SE_T_ of up to 75 dB) with low reflectance (SE_R_ of 1.5 dB at the resonant frequency) owing to EMI absorption induced by multiple internal reflection generated in the LM grid architectures. Remarkably, the excellent stretchability of the LM-based devices facilitates tunable EMI shielding abilities through grid space adjustment upon strain (resonant frequency shift from 81.3 to 71.3 GHz @ 33% strain) and is also capable of retaining shielding effectiveness even after multiple strain cycles. This newly explored device presents an advanced paradigm for powerful EMI shielding performance for next-generation smart electronics.

**Supplementary Information:**

The online version contains supplementary material available at 10.1007/s40820-024-01457-7.

## Abstract (highlights)

- Multiple internal reflection-based absorption-dominant stretchable electromagnetic shielding thin film by incorporating liquid metal grid structure is developed.
- The device demonstrates high electromagnetic shielding effectiveness (SE) (SE_T_ of up to 75 dB) with low reflectance (SE_R_ of 1.5 dB at the resonant frequency).
- The shielding properties of the device can be tuned by adjusting the liquid metal patterned grid spaces upon strain.

**Supplementary Information:**

The online version contains supplementary material available at 10.1007/s40820-024-01457-7.

## Graphical abstract

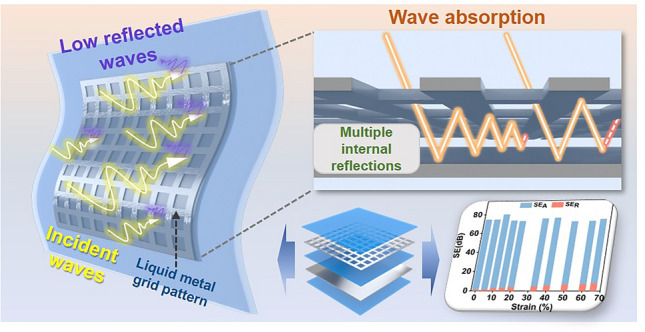

## Introduction

**Highlights**

Recently, intelligent electronic devices have been growing rapidly to meet the demand in various fields ranging from communication facilities and wireless networks to portable and wearable hardware. However, the adverse effect encountered as a result of escalated use of these devices is the rise in electromagnetic pollution arising from electromagnetic interference (EMI), which not only threatens the functionality and durability of electronic devices, but also affects several biological processes in living beings [1–3]. As a result, development of EMI shielding materials has received a tremendous boost [4–10]. Specially, EMI shields fabricated out of thin film materials are the current research hotspots that aim to match the trend of uprising conformal and highly integrated compact electronics, ranging from wearable devices to high-end military and aerospace applications [11, 12]. At the same time, the need for real-time adjustable shielding performance to address intelligent application requirements like passing serviceable EM waves [13], information leakage suppression [14, 15], etc., has led to growing demand of engineering of EMI shielding materials with adjustable shielding properties. Therefore, development of soft, stretchable, and thin film-shaped EMI shielding materials with the ability to modulate shielding abilities can cater the demand of next-generation electronic systems.

While conventional metals and their alloys can function effectively as shielding materials due to their superior electrical conductivity, inadequate resistance to corrosion and limited deformability constrain their applicability in EMI shielding [16, 17]. Additionally, high reflectivity of metals leads to secondary electromagnetic pollution [17]. In lieu of the use of metals, polymer-based EMI shielding materials, with various conductive fillers such as graphene, carbon fibers, carbon nanotubes, and MXenes arranged into segregated structures or preferred orientations within the polymeric matrices, are widely used owing to their distinctive attributes like exceptional corrosion resistance, adequate deformability for application in conformal and wearable electronics, and tunable EMI shielding performances such as modulating the shielding effectiveness, and resonant frequency. [11, 18–24]. Additionally, the filler properties like surface morphology, aggregation, alignments, etc., can be specifically tuned to target electromagnetic waves in different bands or frequency ranges [25–28]. Recently, efforts have been made to develop such polymer-particle-based hybrid EMI shielding materials with low reflectivity through multiple internal reflections [29, 30], but in most cases, low reflectivity is simultaneously associated with low absorptivity of the conducting materials [31, 32]. While MXenes, a class of two-dimensional conductive materials, exhibit enhanced absorption efficiency, their oxidation-induced degradation under environmental conditions significantly limits their potential for practical applications [33, 34]. At the same time, incorporating these fillers to achieve higher shielding performances either requires higher filler to polymer ratio or micro-level engineering for specific filler orientations, which often makes fabrication and processing steps challenging. Moreover, their repeated deformations may degrade overall electro-mechanical properties of the composites due to various factors like misorientation of the trapped fillers, debonding at the polymer-filler interfaces, micro-crack initiation and propagation, etc., thereby threatening their longtime usage [21].

To address the aforementioned issues, it is not only necessary to explore conductive materials that can effectively shield under deformation, but also deduce methodologies that can utilize these materials to achieve low reflectivity-based EMI shielding functionality. For the first case, liquid metal (LM), a promising candidate due to its commendable electrical conductivity and fluidity [35–43], has recently emerged as the forefront material for development of soft and stretchable EMI shielding materials [44–48]. However, given its metallic properties, this class of materials still exhibit high reflectivity. As a solution to the second case, foam structures have been widely used as effective absorption-dominant materials, but their high void content increases their thickness [49, 50]. A few recent studies suggest an alternative approach of usage of conductive grid patterns to achieve low reflection and high absorption at specific frequencies where the minimum reflection is achieved at the resonant frequency on matching the wavelength of the electromagnetic wave (EMW) with the grid period [24, 29]. This method is quite promising in terms of significantly reducing the size of the device, however, certain challenges persist, viz., poor choice of materials that can challenge the structural stability for prolonged use and time-intensive, optimization-critical printing technologies.

This study presents a soft and stretchable thin-film-shaped liquid metal grid-patterned device (LMGD) for effective EMI shielding, featuring low reflectivity and superior absorption-dominant shielding effects. The LMGD, comprising a liquid metal layer and grid pattern separated by an elastomer layer, achieved high electromagnetic shielding effectiveness (SE) up to 75 dB due to multiple internal reflection-induced absorption behavior. The LM grid structure is obtained via aerosol deposition of LM using an airbrush through recyclable OHP line-stencils, offering rapid and cost-effective fabrication compared to conventional printing methods. With minimal electromagnetic wave reflection (SE_R_ < 1.5 dB at resonant frequency) determined by the LM grid geometries, the LMGD’s stretchable and highly elastic properties enabled tunable shielding abilities by adjusting grid spacing under strain. Notably, at a strain value of 10%, the resonant frequency recorded 77 GHz, which corresponds to automotive radar band used in self-driving cars with ADAS (Advanced Driver Assistance System) [51, 52]. Additionally, it exhibits excellent shielding retention after multiple strain cycles, attributed to the preserved continuity of embedded LM structures. To the best of our knowledge, this study reports a significant advancement in the field of EMI shielding devices, exhibiting the highest performances of SE in terms of low reflection, high absorption properties, and SE stability and retention maintained after repeated external deformations.

## Experimental Section

### Materials

Eutectic Gallium Indium alloy (EGaIn) was obtained from Indium Corporation, USA. DragonSkin 10 medium, an ultrastretchable two-component elastomer, was obtained from Hyup Shin, Korea. OHP sheets for preparing stencils were obtained from Coupang, Korea. All the samples were casted in laboratory-scale square-shaped petri dishes.

### Fabrication of LMGD

**Fig. 1:**
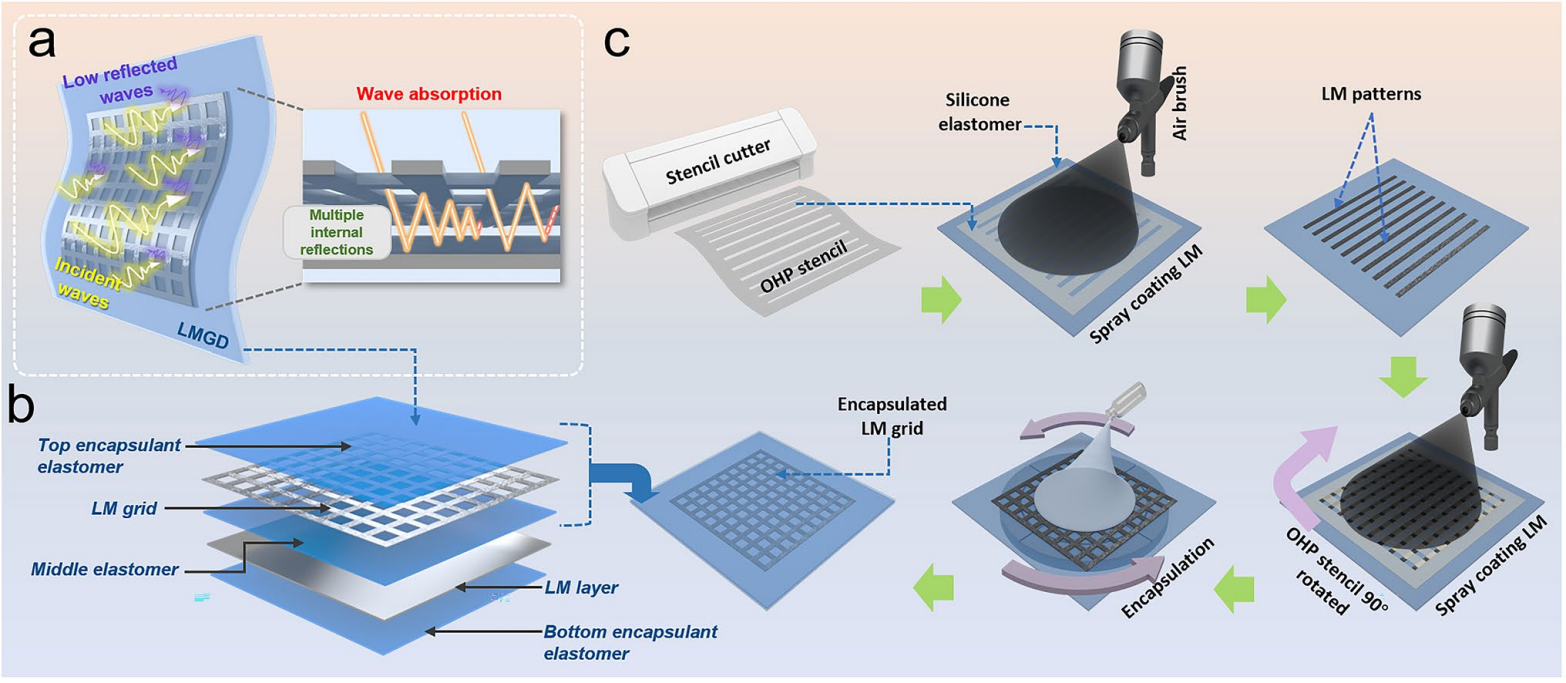
**a** Schematic diagram illustrating the concept of maximization of incident EM wave absorption with lower reflected waves through multiple internal reflections in LMGD. **b** Schematic diagram showing the different components of the designed LMGD. **c** Schematic diagram illustrating the grid architecture fabrication using two-step spray coating of LM on a stretchable and elastic substrate through recyclable OHP stencil

Uncured silicone elastomer was first prepared by mixing components A and B in a 1:1 weight ratio in a paper cup. After thorough stirring, the resulting mixture was spin-coated onto an acrylic plate (10 cm × 10 cm, coated with Ease-Release, a general-purpose releasing agent) using a spin coater (Dongah Trade Corp ACE-200) at 1000 rpm for 90 s. Subsequently, the spin-coated elastomer layer was cured in an oven at 100 °C for 5 min to render a thin, semi-cured silicone layer. The OHP film stencil (designed using Silhouette Cameo 4), consisting of parallel negative spaces, was positioned precisely on the elastomer film, and EGaIn was evenly distributed on the surface using an airbrush (Monster Brush 001) by maintaining a distance of 15 cm from the substrate. The stencil was then rotated by 90° and LM was re-spray-coated to obtain the grid design. Finally, uncured silicone was spin-coated after removing the stencil and subsequently cured to produce the LM grid elastomer film. To obtain the secondary LM layer, another stencil consisting of negative space equivalent to the desired area was used to spray coat LM on the first film and then encapsulated by elastomer, realizing the final device. The detailed process is illustrated in Fig. 1 along with its technical aspects and advantages in the introduction section. For EMI SE measurements, square samples having dimensions 10 cm × 10 cm were prepared, whereas for mechanical and electrical characterizations, smaller samples of 2.5 cm × 2.5 cm were prepared to fit the grippers of universal testing machine.

### Characterization

The EMI SE measurements were carried out using Keysight N5291A vector network analyzer assisted by a free space measurement system (EMLabs FS-110). All resistance-related measurements were carried out using benchtop multimeter (Keysight 34461a). The mechanical properties of the samples were assessed using a universal testing machine (Quasar 2.5 single column). Tilting and adhesion test images were captured using a Phoenix 300 tilting contact angle measurement system, and all microscopic images were captured using an Olympus CX23 instrument.

### Measurement of EMI SE

The network analyser method was used in this study for far-field EMI SE measurements. The EMI shielding effectiveness in the far field can be calculated by measuring the scattering parameters (S_11_, S_21_, S_22_, S_12_), based on which reflectance ($R$), transmittance ($T$), and absorbance ( $A$) were calculated according to the following equations [53, 54]:

1 $$\begin{array}{c}R={\left|{S}_{11}\right|}^{2}={\left|{S}_{22}\right|}^{2}\end{array}$$

2 $$\begin{array}{c}T={\left|{S}_{12}\right|}^{2}={\left|{S}_{21}\right|}^{2}\end{array}$$

3 $$\begin{array}{c}A=1-R-T\end{array}$$

Total shielding effectiveness SE_T_, reflective shielding effectiveness SE_R_, and absorptive shielding effectiveness SE_A_ can be calculated using T, R, and A, respectively, according to the following equations:

4 $$\begin{array}{c}{\text{SE}}_{\text{T}}=10\text{log}\left(\frac{1}{T}\right)=10\text{log}\left(\frac{1}{{\left|{S}_{12}\right|}^{2}}\right)\end{array}$$

5 $$\begin{array}{c}{\text{SE}}_{\text{R}}=10\text{log}\left(\frac{1}{1-R}\right)=10\text{log}\left(\frac{1}{{1-\left|{S}_{11}\right|}^{2}}\right)\end{array}$$

6 $$\begin{array}{c}{\text{SE}}_{\text{A}}=10\text{log}\left(\frac{1-R}{T}\right)=10\text{log}\left(\frac{{1-\left|{S}_{11}\right|}^{2}}{{\left|{S}_{12}\right|}^{2}}\right)\end{array}$$

7 $$\begin{array}{c}{SE}_{T}={SE}_{R}+{SE}_{A}\end{array}$$

## Results and Discussion

### LMGD Designing

Figure 1a illustrates the proposed concept of using an LM grid and an LM layer to maximize the absorption of incident EM waves through multiple internal reflections within the device. As shown in Fig. 1b, the LM grid and the LM layer are separated by an elastomeric layer that acts as a physical separator between the LM grid and the LM layer and facilitates space for the multiple internal reflection to occur. The grid structure is capable of acting as an inductive filter circuit, allowing EM waves of specific wavelengths ideally matching with grid period to pass through it [29, 55], while encountering reflection in the LM layer behind to enable multiple internal reflections or scattering within the device between the grid and layer. Figure 1c describes the overall fabrication process of LMGD film-type EMI shielding device. In order to guarantee both safety and durability of the device for prolonged utility and the ability to adapt to stretching of various degrees, DragonSkin—an ultrastrechable silicone—was chosen as the matrix elastomer due to its exceptional stretchability and easy processing. In order to embed desired LM designs in the elastomer, there are number of methods to choose from that have emerged in the recent years. Although replica molding followed by plasma bonding for fabricating soft microfluidic channels in rigid silicones, followed by injection of LM through them is a commonly employed technique to obtain stretchable and conductive LM patterns, the method encounters several challenges when applied to materials like soft and stretchable silicones (e.g., DragonSkin or Ecoflex) [56]. Silicone oils present in such soft silicones can migrate to the surface during plasma treatment, causing interference with the plasma bonding process [57]. Moreover, injecting LM poses a risk of damaging the device due to the injection hole, potentially compromising its mechanical properties. At the same time, high pressures required to fully inject LM cannot be sustained by the thinness and the intrinsic low elastic modulus of the elastomers. Therefore, a modified lithography-free method to fabricate liquid metal designs on the silicone elastomer was adopted that followed a two-step spray coating of LM through a recyclable OHP stencil using an LM filled airbrush (Fig. 1c). This process can not only substantially reduce LM design printing time but can also help in recycling of deposited LM on the positive space of stencils [58]. As the target LM design is a grid structure, the corresponding ideal stencil would consist of numerous unconnected islands to generate the negative space through which the LM is supposed to be spray coated (Fig. S1). However, in practical case, attaining such stencil designs is impossible, as the positive components of such a stencil will have no physical connections with each other and the stencil frame. In order to address this problem, the stencil is designed in the form of parallel positive and negative spaces, and by spray coating the LM through the stencil by rotating at 90° to the initial deposited LM patterned on the substrate, intersecting LM grid patterns can be generated. This feat was possible due to the superior surface adhesion of LM on elastomer surface over the stencil (discussed later in this section). As the LM exits the airbrush upon release of compressed air, the air flow breaks the LM into small particles that stick on the substrate by virtue of the adhesive oxide layers realizing an LM film. Once the LM grid patterns were formed by removing the stencil, uncured silicone elastomer was spin-coated to encapsulate the LM grid patterns. The adhesion of oxide layer on elastomer prevents the LM design from shifting against the forces during the spin-coating process and the arising friction force from the encapsulating elastomer. The thicknesses of the encapsulating layers can be precisely controlled by manipulating the spin-coating speed, as illustrated in Fig. S2. The film thickness (*h*) can be theoretically predicted using the Meyerhofer model, as represented by the modified equation [59]:

8 $$\begin{array}{c}h=\frac{{h}_{0}}{\sqrt{1+\frac{4\rho {W}^{2}{h}_{0}^{2}t}{3\mu }}}\end{array}$$

where the ${h}_{0}$ is the initial thickness of the coating material. The ultimate thickness of the film is predominantly influenced by the angular velocity and time, with these factors exerting a more substantial impact than other variables. Given that$W=\frac{\pi }{30}\times \varpi$, where the $\varpi$ is the number of revolutions per minute. So, the ultimate thickness of the film can be conceptualized as a bivariate function dependent on both time (*t*) and angular velocity ($\varpi$) [59].

**Fig. 2:**
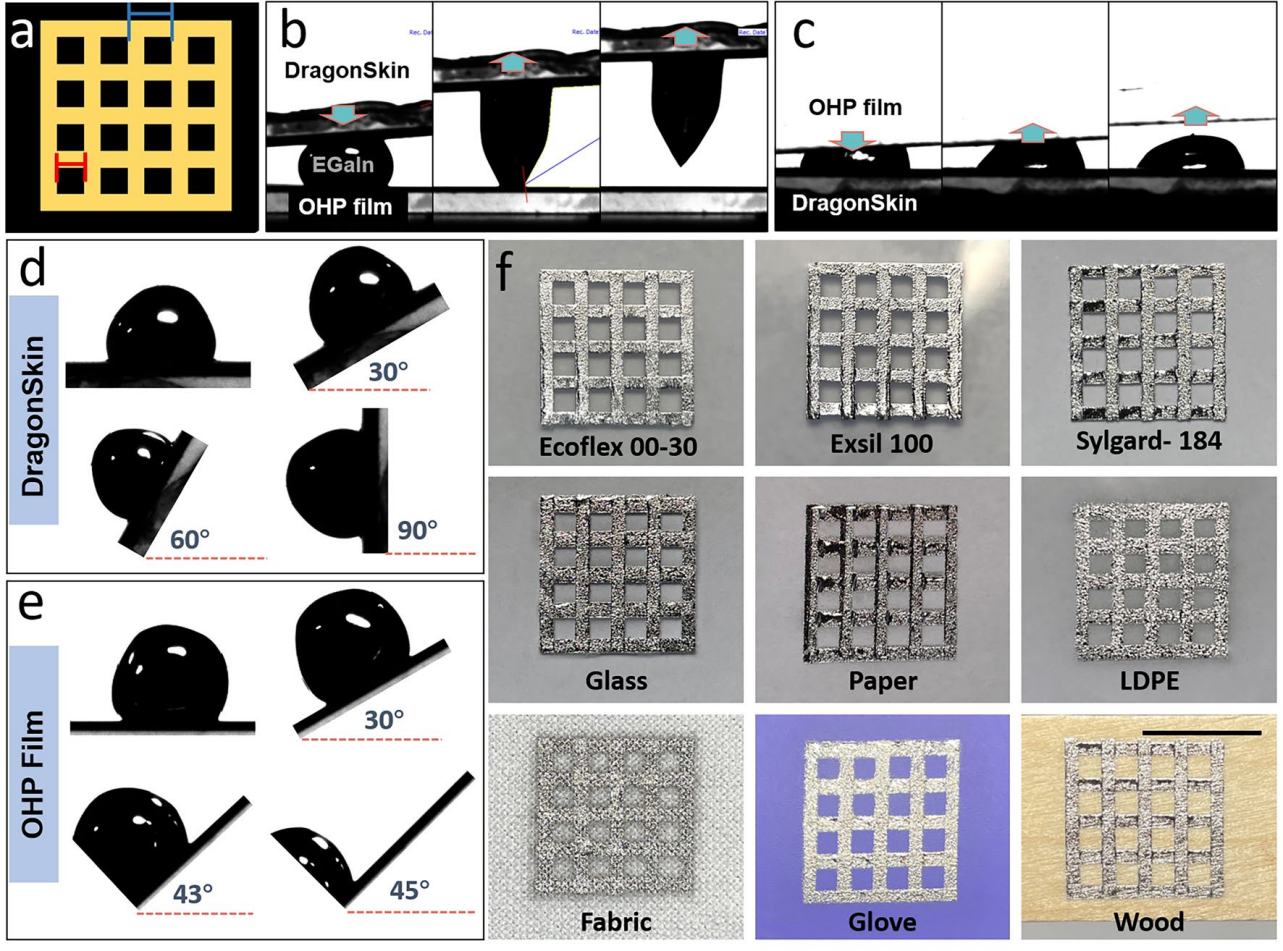
**a** Illustration showing the design parameters of LM grid patterns. The blue and red scales represent grid spacing and grid gap respectively. **b**, **c** Adhesive properties of the LM on DragonSkin (silicone elastomer) and OHP film, respectively. **d**, **e** Tilting angles of LM to initiate sliding on **d** DragonSkin and **e** OHP surfaces. **f** LM grid patterns formed by two-step spray coating on various substrates, including silicones elastomers (Ecoflex 00–30, Exsil 100 and Sylgard-184), glass, paper, low-density polyethylene (LDPE), fabric, nitrile glove, and wood. The scale bar is 1 cm

As described previously, a two-step spray coating process was adopted to form LM grid patterns (Fig. 2a) on various substrates. In order to ascertain the superior adhesion of LM on elastomer over stencil, a simple place-and-pick test was carried where an LM droplet was placed on one’s surface and the other’s surface was brought in contact and removed. As shown in Fig. 2b, c, the LM exhibited low surface adhesion to the OHP film stencil. On comparing the tilting angles initiating slide of LM on the silicone elastomer and OHP film, it was observed that the LM droplet remained on the silicone surface until the tilting angle reached 90° (Fig. 2d). However, the LM droplet began to slide when the tilting angle on the OHP film (Fig. 2e) reached 45°, indicating the inferior adhesion of the LM on the OHP surface. This ensured that when the OHP stencil is removed from the DragonSkin substrate after spray coating LM, the LM adhered exclusively to the substrate’s surface, preserving the integrity of LM patterns. Therefore, the OHP stencil that was used to pattern the first set of parallel lines could be rotated at 90° to spray the second set of parallel lines perpendicular to the first set, resulting in LM grid patterns by a two-step spray coating on various substrates, including silicones (Ecoflex 00–30, Exsil 100, Sylgard-184), glass, paper, low-density polyethylene (LDPE), fabric, nitrile gloves, and wood (Fig. 2f). These grid patterns can also be formed using different methods, including vacuum-assisted filling [60], injection and molding using an elastomeric stamp [61], and direct printing [62]. However, the two-step spray coating of LMs directly forms multi-junctioned LM patterns on various substrates without using any complicated printing or lithographic methods. Spray coating using an airbrush atomizes the LM into small droplets with a thin oxide layer (1–3 nm) on the surface, resulting in a higher surface-to-volume ratio of the oxide layer, compared with the rheologically oxidized LM paste patterned on the surface using a brush [63]. This allowed consistent contact of the LM with the silicone substrate without displacement or aggregation, even after spin coating at a rate of up to 3,000 rpm and repeated stretching cycles (Fig. S3). In addition, the thickness of the LM layer can be precisely controlled by manipulating the airbrush–substrate distance and the spray coating duration (Fig. S4). Also, the stencils can be recycled by first removing the LM deposited on the stencil by simply swiping with an aqueous 0.1 M hydrochloric acid solution saturated cotton swab and finally washing the stencil surface with detergent (Fig. S5).

### Mechanical Properties of LMGDs

**Fig. 3:**
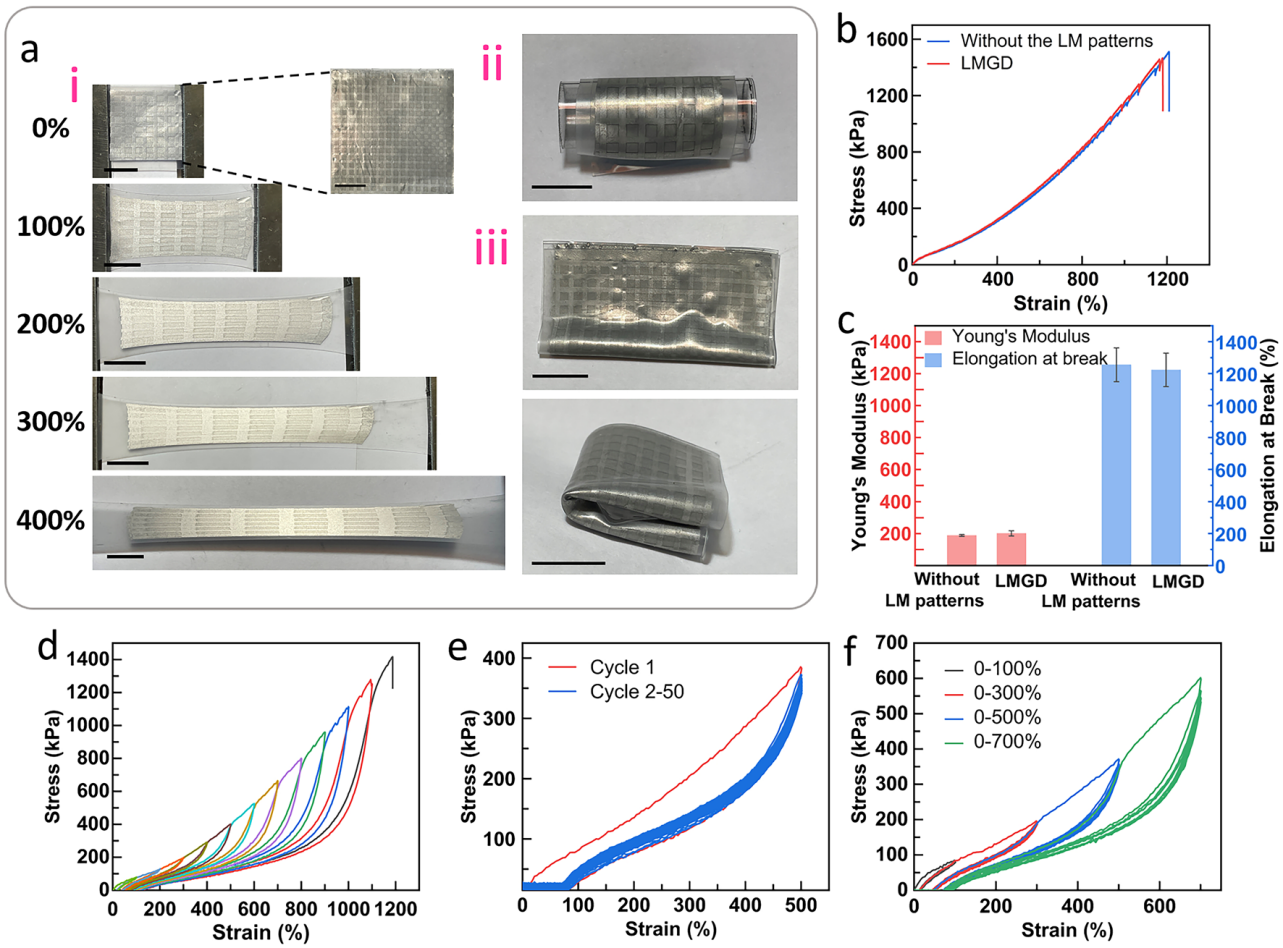
**a** Photographs showing deformability of the LMGD: (i) stretchability (400%), (ii) rollability, and (iii) foldability. The scale bar is 2 cm. **b** Stress–strain curve of the LMGD and silicone elastomer film without the LM patterns. **c** Young’s modulus and elongation at break of the LMGD and silicone elastomer film without the LM patterns. Stress–strain curves of **d** LMGD for progressive tensile strains with single cycle from 0 to 1200% at the intervals of 100%, **e** LMGD during consecutive 50 cycles of strains at 500%, and **f** LMGD for repeated progressive tensile strains with five cycles for strain values of 100%, 300%, 500%, and 700%

In order to achieve multiple internal reflections of incident electromagnetic waves (EMW), a secondary LM layer was stencil printed on the described grid structure and encapsulated (discussed in the next section). To demonstrate high deformability of the LMGDs, ultrastretchable and soft silicone (DragonSkin) with high elongation at break (1000%) and a low modulus (0.5 MPa) was chosen [57]. DragonSkin exhibits remarkable flexibility allowing it to seamlessly conform to diverse surfaces when utilized as a mountable device. Additionally, the incorporation of LM within DragonSkin does not impede the device’s ability to deform, ensuring optimal adaptability during use. Figure 3a–i shows the 410-µm-thick LMGD stretched uniaxially up to 400% by a manual stretcher, where the embedded liquid metal designs were observed to stretch accordingly while maintaining interconnected electrode networks upon strain. Similarly, Fig. 3a-ii,iii show the states of the device in rolled and folded states respectively. Next, the device was subjected to uniaxial stretching in order to compare its mechanical properties with its unpatterned elastomer counterpart. The presence of the LM patterns slightly increased Young’s modulus (from 188.7 ± 5.9 to 201.4 ± 10 kPa) and decreased the elongation at break (from 1255 ± 105% to 1223 ± 104%) (Fig. 3b, c) owing to the stiffening effect from increased oxide layer due to spray coating [64, 65], and fracturing along the pattern respectively. When the strain value of both LMGD and unpatterned film approached near 600%–800%, fine sawtooth patterns could be observed in the stress–strain curve. This phenomenon is presumably due the fact that since the samples have three layers of elastomers, there are two elastomeric interfaces which might have suffered slight microscopic interfacial delamination at very high strain values.

Next, the LMGD was subjected to a series of cyclic loading and unloading tests in order to ascertain its durability and longtime usability. First, as shown in Fig. 3d, the LMGD was subjected to progressing cyclic test involving a step-wise increment 100% strain till break at about 1200%. A few distinct sawtooth patterns were observed at higher strain values suggesting microscopic interfacial delamination. This was followed by 50 cycles of loading–unloading upto 500% and subsequent step-wise cyclic tests involving five cycles each till 700% of another identical LMGD sample (Fig. 3e, f). As seen in the graphs, the LMGD was elastically reversible due to minimal changes in the silicone polymeric network and rheological properties of the LM during strains. After the cycling tests, the LMGD exhibited a slight longitudinal deformation close to 5% (Fig. S6) occurring during the initial loading of the strain on the sample leading to some irreversible plastic deformation that can be attributed to the Mullins effect.

### EMI Shielding Performance of LMGDs

**Fig. 4:**
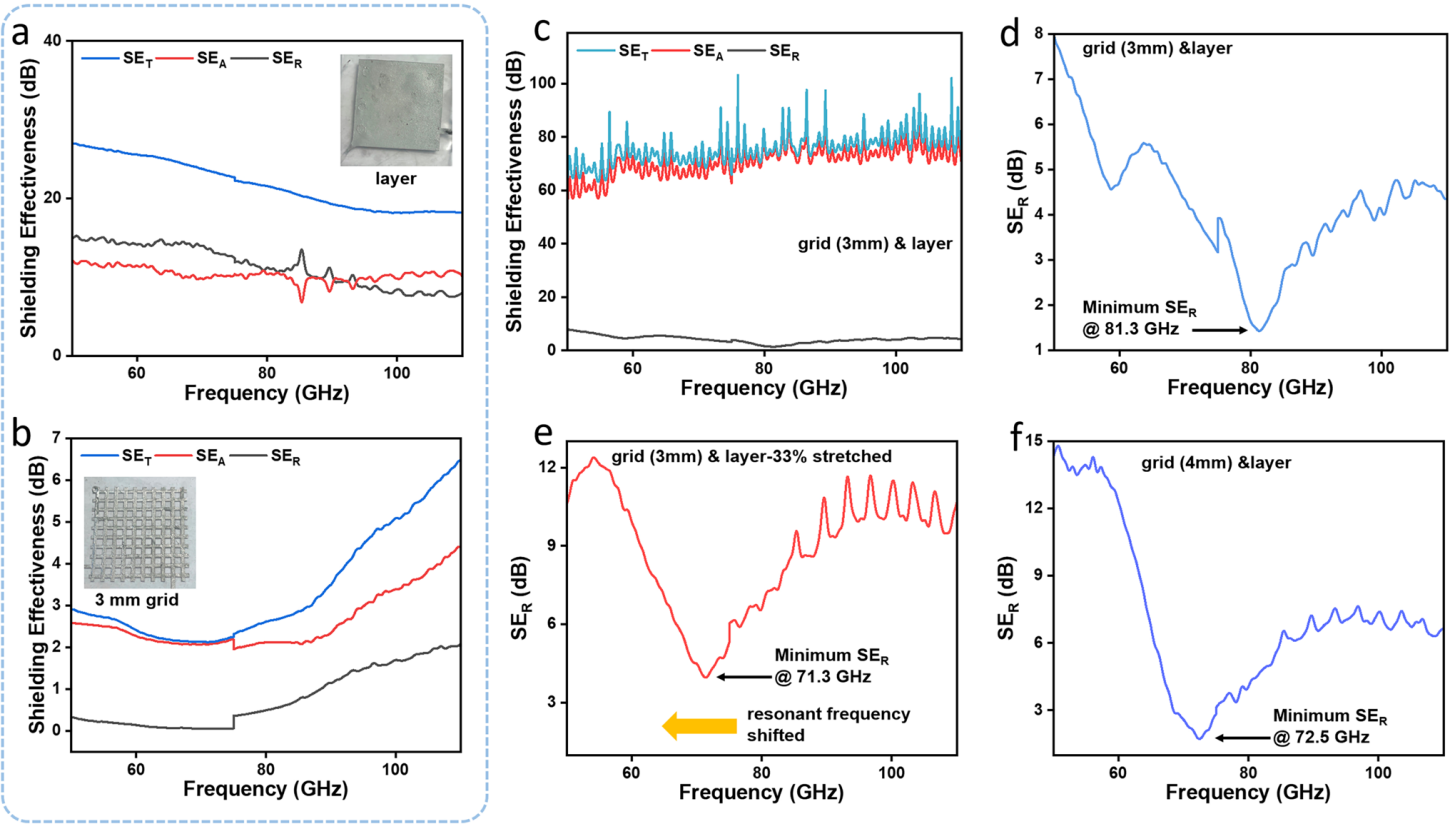
**a** EMI (SE) of LM layer for EMWs at 50–110 GHz. **b** EMI (SE) of LM grid pattern with a grid spacing of 3 mm for EMWs at 50–110 GHz.  **c** EMI (SE) and **d** reflection (SE_R_) of the 410-µm-thick LMGD with a grid spacing of 3 mm for EMWs at 50–110 GHz. **e** SE_R _of the 410-µm-thick LMGD with a grid spacing of 3 mm when subjected to a strain of 33% for EMWs at 50–110 GHz. **f** SE_R_ of the 410-µm-thick LMGD with a grid spacing of 4 mm for EMWs at 50–110 GHz

Considering the fact that due to the extreme deformability of both LM and elastomer, LM-patterned devices can exhibit good metallic conductivity even under strain [66–68], the LMGD was utilized as a soft and stretchable EMI shielding material. As illustrated in Fig. 1b, the device consisted of three elastomer layers, out of which the top and bottom elastomer layers are meant for device encapsulation while the middle elastomer acts as the physical barrier between the LM layer and the LM grid pattern. This multilayered structure can effectively shield against EMI through multiple reflections of the EMWs within the device. In order to understand the shielding properties of the individual LM layers, the (SE) of both layers was tested. The LM thin layer exhibited a high EMI (SE_T_) more than 20 dB (Fig. 4a), whereas the LM grid patterns resulted in near-zero (SE_R_) at the resonant frequency (Fig. 4b). The phenomenon is pictorially depicted in Fig. S7. Thus, by combining these layers, the EMWs are transmitted through the elastomer between the LM grid patterns but are reflected and absorbed by the thin LM layer in the polymer matrix by the multiple reflection and absorption between the two LM layers. The LMGD with a grid spacing of 3 mm showed a high shielding capability (SE_T_ of 75 dB) at frequencies ranging from 50 to 110 GHz (Fig. 4c), owing to the multiple reflections and absorption of the EMWs between the layers. In particular, the minimum SE_R_ was only 1.4 dB at a frequency of 81.3 GHz (Fig. 4d), which corresponds well with the results of theoretical calculations (Note S1 and Table S1).

In addition, the grid spacings of LMGD can be manipulated upon stretching the device in the direction parallel to the electrodes. Using the LMGD with an initial grid spacing of 3 mm, when a strain value of 33% was applied to the device, shifting in the resonant frequency occurred from 81.3 to 71.3 GHz (Fig. 4e). Theoretically, at this strain value, the grid spacing should increase to 4 mm; however, under practical cases, the width of the lines of LM grid would also stretch by an identical strain value. To verify the consistency of the resonant frequency shifting, SE_R_ of another LMGD with an initial grid spacing of 4 mm was measured whose resonant frequency was found to be 72.5 GHz (Fig. 4f) which is only 1.68% higher than the value obtained when the LMGD with 3-mm grid spacing was stretched to 33%. This ability to tune the resonant frequency upon stretching enables the LMGD to be utilized as a stretchable electromagnetic shielding material that can effectively function as a frequency pass filter for EMWs [55, 69].

**Fig. 5:**
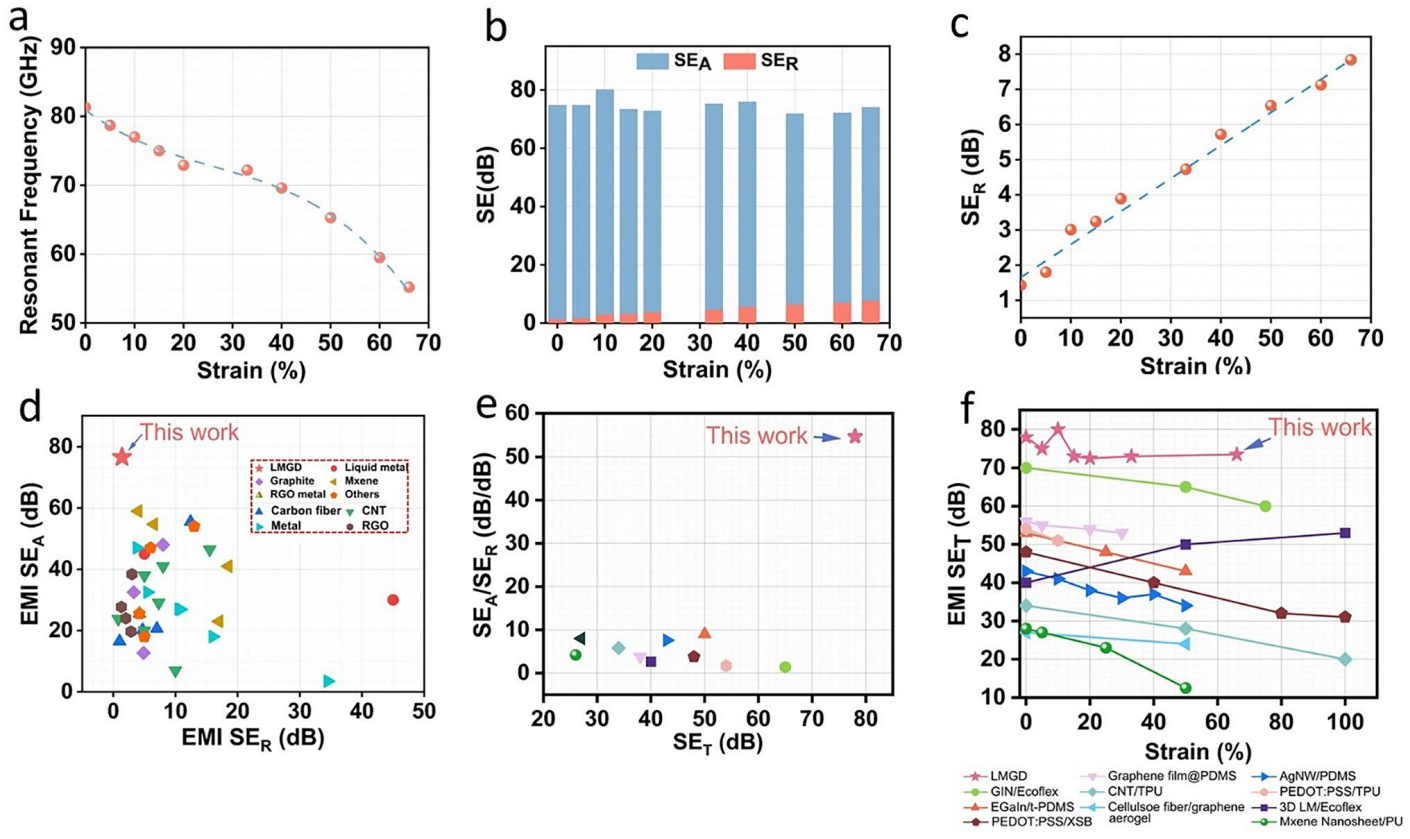
**a** Comparative analysis of resonant frequencies of the LMGD subjected to tensile strain from 0 to 66%. **b** EMI SE, including SE_R_ and SE_A_ of the LMGD, averaged over frequencies 50–110 GHz at strains of 0–66%. **c** EMI SE_R_ of the LMGD with a grid spacing of 3 mm under the loading tensile strain of up to 66%. **d** Comparison of SE_A_ of various EMI shielding materials, including metals (Ni-Co, Ag, and Cu nanowires) and others (carbon aerogel, Fe_2_H_2_NiO_4_, and Fe_2_O_3_), as a function of SE_R_. **e** Comparison of the SE_A_/SE_R_ of various stretchable EMI shieling materials as a function of SE_T_. **f** Comparison of the SE_T_ of various stretchable EMI shieling materials as a function of strain. The results were collected from previous studies, as shown in Tables S2–S4

To investigate the effects of mechanical stretching of the device on EMI shielding effectiveness (SE) and corresponding resonant frequencies, the LMGD was subjected to a strain of up to 66%, and the corresponding EMI shielding properties were measured. As illustrated in Fig. 5a, the resonant frequency decreased monotonically with increasing strain, whereas SE_T_ remained at a high average of 75 dB (Fig. 5b) which is well match with the theoretical results (Table S1). There was a slight increase in the SE_R_ and a corresponding decrease in the SE_A_ while the strain increased, presumably owing to the decreased cross-sectional area of the polymer matrix during stretching. Upon strain, the EMW reflected from the LM layers directly propagated backward to the EMW source, resulting in a lower SE_A_ and a higher SE_R_ for the LMGD (Fig. 5c). Interestingly, at a strain value of 10%, the resonant frequency recorded 77 GHz, which corresponds to automotive radar band used in self-driving cars with ADAS [51, 52]. Thus, it can be inferred that the shielding properties of the LMGD can be tuned by mere stretching to meet a certain target resonant frequency.

To investigate the durability of the LMGD, changes in the SE and effective resistance upon repeated loading and unloading tensile strain of 100% over 100 cycles were monitored (Fig. S8). The average (SE_T_) after 100 cycles of strain was approximately 70 dB, which is similar to that of LMGD prior to the application of the tensile strain (Fig. S8a, b), owing to preserved geometries of the electrodes even after cycles of strain. However, repeated loading and unloading strains could cause the oxide layer to break and reform; thus, it may accumulate on the LM surface, resulting in a thicker oxide layer and increased effective resistance (Fig. S8c, d).

Figure 5d compares the SE_A_ and SE_R_ values of LMGD with previously reported studies. To the best of our knowledge, the LMGD developed in this work exhibits the best SE in terms of low reflection and high absorption properties reported to date. A detailed comparison of the various materials used along with thickness of the fabricated devices as well as the shielding properties is given in Table S2. Although previous studies on EMI shielding have mostly focused on improving the SE_A_ without considering the reduction in the SE_R_, high reflection can act as a source of EM pollution that interferes with other components. As shown in Fig. 5e, LMGD exhibited the highest SE_A_to SE_R_ ratio (54.7) and SE_T_ (78 dB) when compared to previous studies on stretchable EMI shielding materials. A detailed comparison of the shielding properties of these stretchable devices with LMGD is provided in Table S3. Additionally, Fig. 5f compares the SE_T_ of LMGD with those of previously reported stretchable EMI shielding materials when subjected to strain. While most materials exhibit a decrease in SE_T_ upon straining, LMGDs can maintain high SE_T_ stability even under large external deformations. The corresponding table of comparison of SE_T_ of these materials along with their reported strain values is given in Table S4. As a practical demonstration (Fig. S9), the LMGD can also effectively shield the EMWs emitted from the Tesla coil, thereby preventing the flow of induced currents and deactivating the light. However, the applicability of such grid structures is not only limited to EMI shielding but can also be extended to other applications, e.g., biomechanical sensing (Fig. S10).

## Conclusions

In this work, soft and stretchable electromagnetic interference shielding thin film device (LMGD) is developed by leveraging stretchable and soft behaviors of both liquid metal and silicone elastomer. The LMGD could achieve high EMI shielding effectiveness (SE_T_ up to 75 dB) with high absorption and low reflectance (SE_R_ of 1.5 dB at the resonant frequency) owing to multiple internal reflections in the frequency range of 50–110 GHz by virtue of strategically patterned LM grid design. By taking advantage of higher surface adhesion of LM on elastomer, the grid design could be obtained by a dual-step aerosol deposition process through recyclable stencils, which is both rapid and cost-effective compared to prevalent printing techniques. The stretchable and elastic properties of the device facilitated strain-induced adjustment of grid spacings, resulting in a shift in the resonant frequency, thereby making it possible to achieve strain-tunable EMI shielding abilities. On stretching the LMGD to 10%, the resonant frequency shifted to 77 GHz, which corresponds to automotive radar band used in self-driving cars with ADAS. The LMGD could also retain its shielding properties even after multiple strain cycles, proving its durability and longtime usability. This thin film-shaped LMGD along with its ability to tune EMI shielding properties would be utilized as a powerful EMI shielding material for the next-generation electronic devices.

## Supplementary Information

Below is the link to the electronic supplementary material.

Supplementary file1 (DOCX 7962 KB)
