# Supplementary material for: Liquid Metal Grid Patterned Thin Film Devices Toward Absorption-Dominant and Strain-Tunable Electromagnetic Interference Shielding
Source: Nanomicro Lett. 2024 Jul 17;16:248. doi: 10.1007/s40820-024-01457-7 (PMC11255180; doi:10.1007/s40820-024-01457-7)
Supplement: Supplementary file 1 — Supplementary file1 (DOCX 7962 KB) [file 40820_2024_1457_MOESM1_ESM.docx]

Supporting Information for

Liquid Metal Grid Patterned Thin Film Devices Toward Absorption-Dominant and Strain-Tunable Electromagnetic Interference Shielding

Yuwen Wei^1, #^, Priyanuj Bhuyan^2, #^, Suk Jin Kwon^3^, Sihyun Kim^1^, Yejin Bae^1^, Mukesh Singh^2^, Duy ThanhTran^1^, Minjeong Ha^4^, Kwang-Un Jeong^1^, Xing Ma^5^, Byeongjin Park^3,^ *, and Sungjune Park^2,^ *

^1^ Department of Nano Convergence Engineering, Jeonbuk National University, Jeonju 54896, Republic of Korea

^2^ School of Chemical Engineering, Sungkyunkwan University (SKKU), Suwon 16419, Republic of Korea

^3^ Composites Research Division, Korea Institute of Materials Science, Changwon 51508, Republic of Korea

^4^ Department of Materials Science and Engineering, Gwangju Institute of Science and Technology, Gwangju 61005, Republic of Korea

^5^ School of Materials Science and Engineering, and Sauvage Laboratory for Smart Materials, Harbin Institute of Technology (Shenzhen), Shenzhen, Guangdong, 518055, China

*^#^* Yuwen Wei1 and Priyanuj Bhuyan contributed equally to the work

*Corresponding authors. E-mail: [sungjunepark@skku.edu](mailto:sungjunepark@skku.edu) (Sungjune Park); [b.park@kims.re.kr](mailto:b.park@kims.re.kr) (Byeongjin Park)

**Supplementary Note S1**

**Theoretical calculations for EMI shielding effectiveness**

The expression for reflection of multilayered materials is shown as [S1]:

$$\begin{aligned} {SE}_{R}=20\log\left( \frac{1}{2}\left| 1+\frac{Z_{1}}{Z_{0}} \right| \right)+20\log\left( \frac{1}{2}\left| 1+\frac{Z_{2}}{Z_{1}} \right| \right)+\cdots+20\log\left( \frac{1}{2}\left| 1+\frac{Z_{n+1}}{Z_{n}} \right| \right)\mathrm{dB}\#\left( S1 \right) \end{aligned}$$

Where Z_n_ is the impedance of each material. For the LM-based multilayered materials, the electrical conductivity of silicone rubber, liquid metal, and the air is around 5×10^-12^ S/m, 6 ×10^4^ S/m, and 8×10^-15^ S/m, respectively, The electrical conductivity of air is much smaller than that of the liquid metal, thus this high impedanc. mismatch results in the LMGDs taking the main contribution of reflection. The reflection can be expressed as [S2]:

$$\begin{aligned} {SE}_{R}=20\log\frac{Z_{0}}{4Z_{1}}=20\log\left( \frac{\sqrt{\mu_{0}\sigma}}{4\sqrt{2\pi f\mu\varepsilon_{0}}} \right)\mathrm{dB}\#\left( S2 \right) \end{aligned}$$

Where $f$, $\sigma$, and $\mu$, are represented as frequency, electrical conductivity, and magnetic permeability of the materials, respectively. The electrical conductivity, magnetic permeability, and vacuum permittivity ($\varepsilon_{0}$) of liquid metal are calculated to be 3.3 ×10^6^ S/m, 0.8 ×10^-6^ H/m and 8.854 × 10^-12^ F/m [S3], respectively. The above equation is applicable only if Z_0_^2^ ($\mu/\varepsilon_{0}$) >> Z_1_^2^ ($2\pi f\mu/\sigma$), equal to $\sigma$ >> $2\pi f\varepsilon_{0}$. For the frequency from 50 to 110 GHz, the largest value of $2\pi f\varepsilon_{0}$ is 6.12 Hz F/m, which is far lower than that of the conductivity of the liquid metal electrode (3.3 ×10^6^ S/m). To verify the reflection at resonant frequency (81.3 GHz) in the experimental results, the theoretical calculation follows:

$${SE}_{R}=20\log(\frac{\sqrt{0.8\times{10}^{-6}\times3.3\times{10}^{6}}}{4\sqrt{2\times\pi\times81.3\times{10}^{9}\times0.8\times{10}^{-6}\times8.854\times{10}^{-12}}})\mathrm{dB}=46.5 \mathrm{dB}$$

The absorption of multilayered EMI shielding materials can be expressed as [S4]:

$$\begin{aligned} {SE}_{A}=8.686d\sqrt{\pi f\sigma\mu} \mathrm{dB}\#\left( S3 \right) \end{aligned}$$

As the permittivity of DragonSkin from 50 to 80 GHz is 2.8, the Maxwell’s Equations can show:

$$\begin{aligned} \lambda_{0}=\frac{c_{0}}{f} (free space)\#\left( S4 \right) \end{aligned}$$

$$\begin{aligned} \lambda=\frac{c_{0}}{f\sqrt{\varepsilon_{r}}} (dielectric)\#\left( S5 \right) \end{aligned}$$

Where $c_{0}$ is the speed of light in a vacuum, f is frequency, $\varepsilon_{r}$ is the permittivity of DragonSkin.

The match grid of each state after stretching can be calculated as shown in Table S1, which is well matched with the actual grid spacing

**Supplementary Tables**

**Table S1** The theoretical and actual matching grid measurements

| Strain (%) | SE_R_(dB) | Freq(GHz) | Matching grid (mm) | Matching grid- considering refractive index (mm) | Actual grid gap (mm) |
| --- | --- | --- | --- | --- | --- |
| 0 | 1.43 | 81.3 | 3.690037 | 2.205219 | 2 |
| 5 | 1.8 | 78.7 | 3.811944 | 2.278072 | 2.1 |
| 10 | 3.01 | 77 | 3.896104 | 2.328367 | 2.2 |
| 15 | 3.24 | 75 | 4 | 2.390457 | 2.3 |
| 20 | 3.89 | 72.9 | 4.115226 | 2.459318 | 2.4 |
| 33 | 3.98 | 71.23 | 4.211709 | 2.516977 | 2.66 |
| 40 | 5.72 | 69.6 | 4.310345 | 2.575924 | 2.8 |
| 50 | 6.54 | 65.3 | 4.594181 | 2.745548 | 3 |
| 60 | 7.13 | 59.5 | 5.042017 | 3.013181 | 3.2 |
| 66 | 7.84 | 55.2 | 5.434783 | 3.247904 | 3.32 |

**Table S2** EMI SE_R_, SE_A,_ and the ratio of SR_A_-to-SE_R_ of various EMI shielding materials

| Type | Materials | | Thickness | EMI SE | | | SE_A_/SE_R_ | References |
| --- | --- | --- | --- | --- | --- | --- | --- | --- |
|  |  |  | mm | SET (dB) | SEA (dB) | SER (dB) |  |  |
| Liquid metal | EGaIn | DragonSkin | 0.4 | 78 | 76.6 | 1.4 | 54.71 | This work |
|  | Ni +EGaIn | Ecoflex | 1 | 75 | 30 | 45 | 0.67 | [S5] |
|  | EGaIn/t | PDMS | 18.75 | 50 | 45 | 5 | 9.00 | [S6] |
| Carbon fiber | Carbon Fiber/Fe_3_O_4_ | PDMS | 0.7 | 67.9 | 55.47 | 12.43 | 4.46 | [S7] |
|  | Carbon Fiber/Fe_3_O_4_ | Epoxy | 13 | 17.5 | 16.5 | 1 | 16.50 | [S8] |
|  | Carbon Fiber | PP | 3.2 | 24.9 | 20.17 | 4.73 | 4.26 | [S9] |
|  | CNF | ABS | 1.1 | 27.6 | 20.6 | 7 | 2.94 | [S10] |
| CNT | MWCNT | PLLA | 2.5 | 24.6 | 23.8 | 0.8 | 29.75 | [S11] |
|  | MWCNT | PC | 1.85 | 25 | 20 | 5 | 4.00 | [S12] |
|  | MWCNT | PP | 1 | 36.4 | 29.1 | 7.3 | 3.99 | [S3] |
|  | MWCNT | WPU | 4.5 | 49 | 41 | 8 | 5.13 | [S13] |
|  | SWCNT | Epoxy | 1.5 | 16.99 | 6.99 | 10 | 0.70 | [S14] |
|  | CNT sponge | Epoxy | 2 | 44 | 38 | 5 | 7.60 | [S15] |
|  | MWCNT | WPU | 0.5 | 62 | 46.5 | 15.5 | 3.00 | [S16] |
| Graphite | Carbon Black | SEBS | 5 | 17.57 | 12.69 | 4.88 | 2.60 | [S17] |
|  | Graphite | PE | 2.5 | 35.8 | 32.5 | 3.3 | 9.85 | [S18] |
|  | Graphite | ABS | 3 | 56 | 48 | 8 | 6.00 | [S19] |
| MXene | Ti_3_C_2_Tx | PS | 2 | 61.2 | 54.7 | 6.5 | 8.42 | [S20] |
|  | Ti_3_C_2_Tx | Sodium Alginate | 0.008 | 40 | 23 | 17 | 1.35 | [S21] |
|  | Ti_3_C_2_Tx | Foam | 0.06 | 63 | 59 | 4 | 14.75 | [S22] |
|  | Ti_3_C_2_Tx |  | 0.04 | 59.5 | 41 | 18.5 | 2.22 | [S23] |
| Metal | Ni-Co Fiber | PES | 2.9 | 38 | 32.5 | 5.5 | 5.91 | [S24] |
|  | Ag+CF | Epoxy | 2.5 | 38 | 3.5 | 34.5 | 0.10 | [S25] |
|  | Cu nanowire | PS | 0.2 | 34 | 18 | 16 | 1.13 | [S26] |
|  | Ag nanowire | PES | 0.01 | 37.7 | 26.9 | 10.8 | 2.49 | [S27] |
|  | Cu Graphite | PVC | 2 | 50.8 | 47 | 3.8 | 12.37 | [S28] |
|  | Ag Nanowire | PANI | 0.1 | 37.5 | 27 | 10.5 | 2.57 | [S27] |
| RGO | RGO | PEI | 2.3 | 22.5 | 19.6 | 2.84 | 6.92 | [S29] |
|  | RGO | PS | 2.5 | 29 | 27.7 | 1.3 | 21.31 | [S30] |
|  | RGO | WPU | 2 | 26 | 24 | 2 | 12.00 | [S31] |
|  | RGO | PS | 2.5 | 41.4 | 38.4 | 3 | 12.80 | [S32] |
| RGO metal | RGO Fe_3_O_4_ | PANI | 2.5 | 29.7 | 25.5 | 4.2 | 6.07 | [S33] |
| Others | Carbon aerogel |  | 10 | 53 | 47 | 6 | 7.83 | [S34] |
|  | Ni ferrite | PVDF | 2 | 67 | 54 | 13 | 4.15 | [S35] |
|  | Fe_2_O_3_ | PEDOT | 6 | 23 | 18 | 5 | 3.60 | [S36] |

CNF: Carbon nanofiber; PDMS: Polydimethylsiloxane; PP: Polypropylene; ABS: Acrylonitrile butadiene styrene; PLLA: Poly-L-lactic acid; MWCNT: Multi-walled carbon nanotube; RGO: Reduced graphene oxide; SWCNT: Single-walled carbon nanotube; PC: Polycarbonate; WPU: Waterborne polyurethane; SEBS: Styrene-ethylene-butylene-styrene; PVC: Polyvinyl chloride; PS: Polystyrene; PVDF: Polyvinylidene fluoride; PANI: Polyaniline; PEDOT: Poly(3,4-ethylenedioxythiophene); PES: Polyether sulfone; PEI: Polyetherimide

**Table S3** EMI SE_R_, SE_A,_ and the ratio of SR_A_-to-SE_R_ of various stretchable EMI shielding materials

| Materials | | EMI SE | | |  | References |
| --- | --- | --- | --- | --- | --- | --- |
|  |  | SE_A_ (dB) | SE_R_ (dB) | SE_T_ (dB) | SE_A_/SE_R_ |  |
| EGaIn | DragonSkin | 76.6 | 1.4 | 78 | 54.7 | This work |
| Ni + EGaIn | Ecoflex | 38 | 27 | 65 | 1.4 | [S5] |
| EGaIn | t-PDMS | 45 | 5 | 50 | 9 | [S6] |
| Graphene film | PDMS | 30 | 8 | 38 | 3.75 | [S37] |
| CNT | TPU | 29 | 5 | 34 | 5.8 | [S38] |
| Cellulose fiber | graphene aerogel | 24 | 3 | 27 | 8 | [S39] |
| AgNW | PDMS | 38 | 5 | 43 | 7.6 | [S40] |
| PEDOT: PSS | WPU | 34 | 20 | 54 | 1.7 | [S41] |
| 3D LM | Ecoflex | 29 | 11 | 40 | 2.6 | [S42] |
| PEDOT: PSS | XSB | 38 | 10 | 48 | 3.8 | [S43] |
| MXene Nanosheet | PU | 21 | 5 | 26 | 4.2 | [S44] |

TPU: Thermoplastic polyurethane; WPU: Waterborne polyurethane; PU: Polyurethane

**Table S4** Comparison of EMI SE_T_ of various stretchable EMI shielding materials

| Materials | | Strain (%) | SE_T_ (dB) | References |
| --- | --- | --- | --- | --- |
| EGaIn | DragonSkin | 0 | 78 | this work |
|  |  | 5 | 75 |  |
|  |  | 10 | 80 |  |
|  |  | 15 | 73 |  |
|  |  | 20 | 72.5 |  |
|  |  | 33 | 73 |  |
|  |  | 66 | 73.5 |  |
| Ni + EGaIn | Ecoflex | 0 | 70 | [S5] |
|  |  | 50 | 65 |  |
|  |  | 75 | 60 |  |
| EGaIn | t-PDMS | 0 | 53 | [S6] |
|  |  | 25 | 48 |  |
|  |  | 50 | 43 |  |
| Graphene film | PDMS | 0 | 56 | [S37] |
|  |  | 5 | 55 |  |
|  |  | 20 | 54 |  |
|  |  | 30 | 53 |  |
| CNT | TPU | 0 | 34 | [S38] |
|  |  | 50 | 28 |  |
|  |  | 100 | 20 |  |
| Cellulose fiber | graphene aerogel | 0 | 27 | [S39] |
|  |  | 50 | 24 |  |
| AgNW | PDMS | 0 | 43 | [S40] |
|  |  | 10 | 41 |  |
|  |  | 20 | 38 |  |
|  |  | 30 | 36 |  |
|  |  | 40 | 37 |  |
|  |  | 50 | 34 |  |
| PEDOT: PSS | WPU | 0 | 54 | [S41] |
|  |  | 10 | 51 |  |
| 3D LM | Ecoflex | 0 | 40 | [S42] |
|  |  | 50 | 50 |  |
|  |  | 100 | 53 |  |
| PEDOT: PSS | XSB | 0 | 48 | [S43] |
|  |  | 40 | 40 |  |
|  |  | 80 | 32 |  |
|  |  | 100 | 31 |  |
| MXene Nanosheet | PU | 0 | 28 | [S44] |
|  |  | 5 | 27 |  |
|  |  | 25 | 23 |  |
|  |  | 50 | 12.5 |  |

**Supplementary Figures**

**
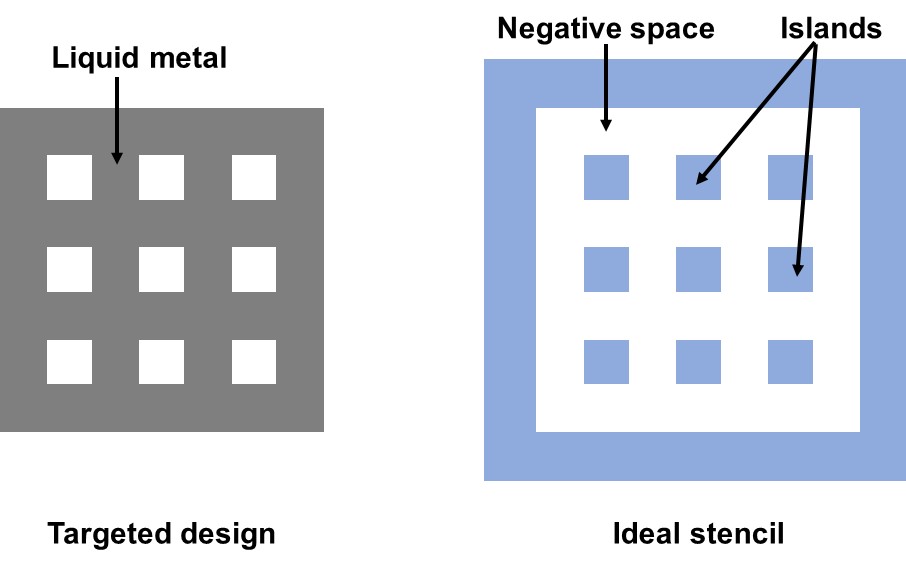
**

**Fig. S1** The target LM grid design and corresponding ideal stencil to achieve the design


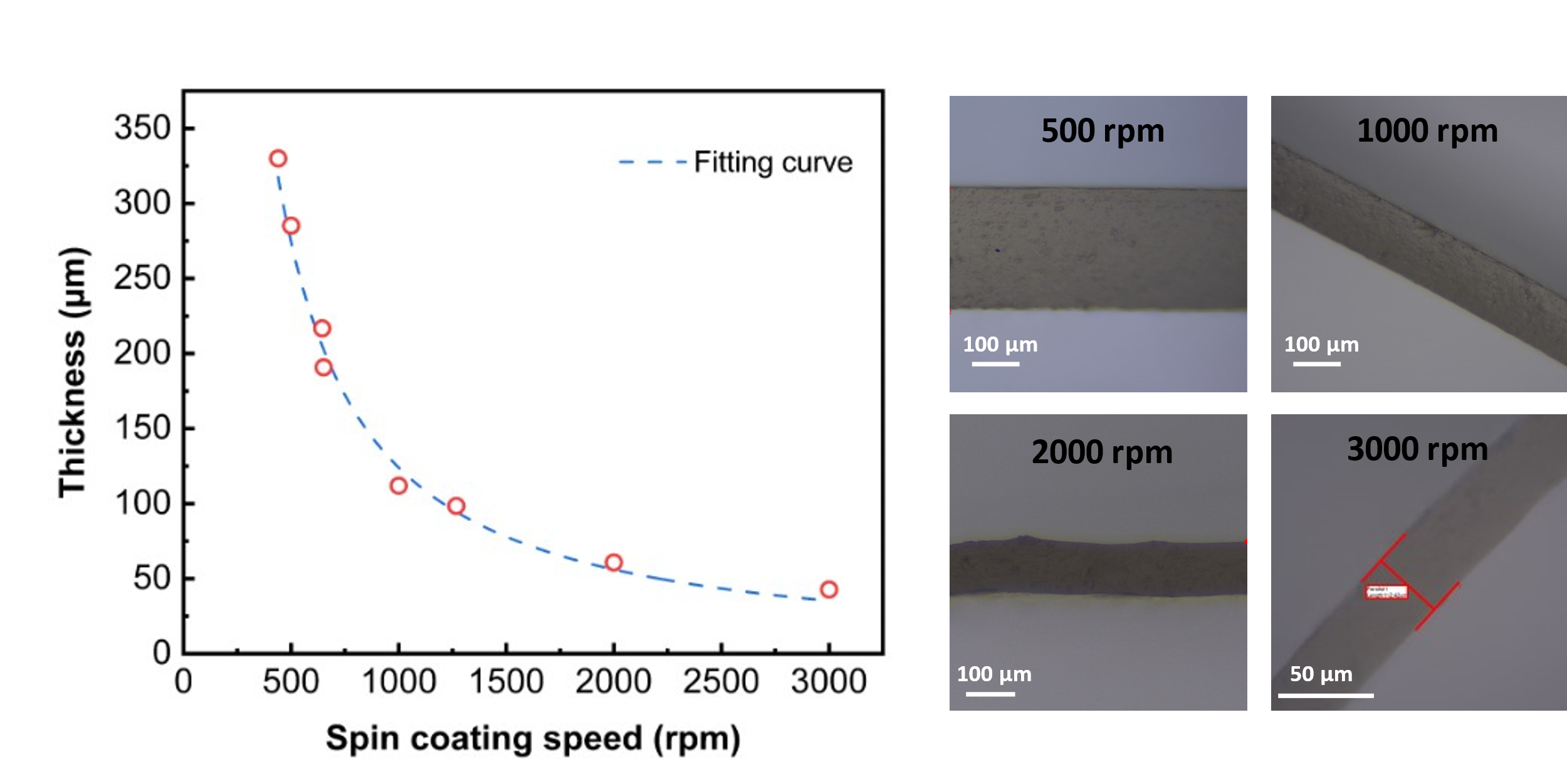


**Fig. S2** The thickness of silicone elastomer films as the function of spin coating speed (left). The optical microscope images of the silicone elastomer films formed by spin-coating at 500 rpm, 1000 rpm, 2000 rpm, and 3000 rpm, respectively (right)

c
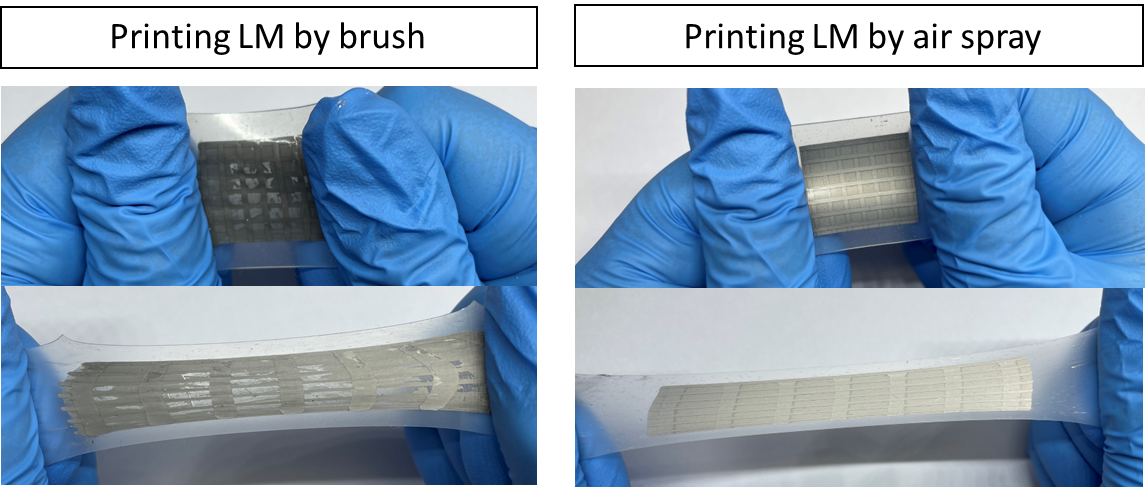


**Fig. S3** Photos showing liquid metal grid patterns formed by (left) printing using a brush and (right) spray coating on a silicone elastomer substrate after multiple stretching. The visible gaps as seen in the first image is due to inability of the brush to properly wet the LM on elastomer surface, while spray coating enables uniform deposition of LM


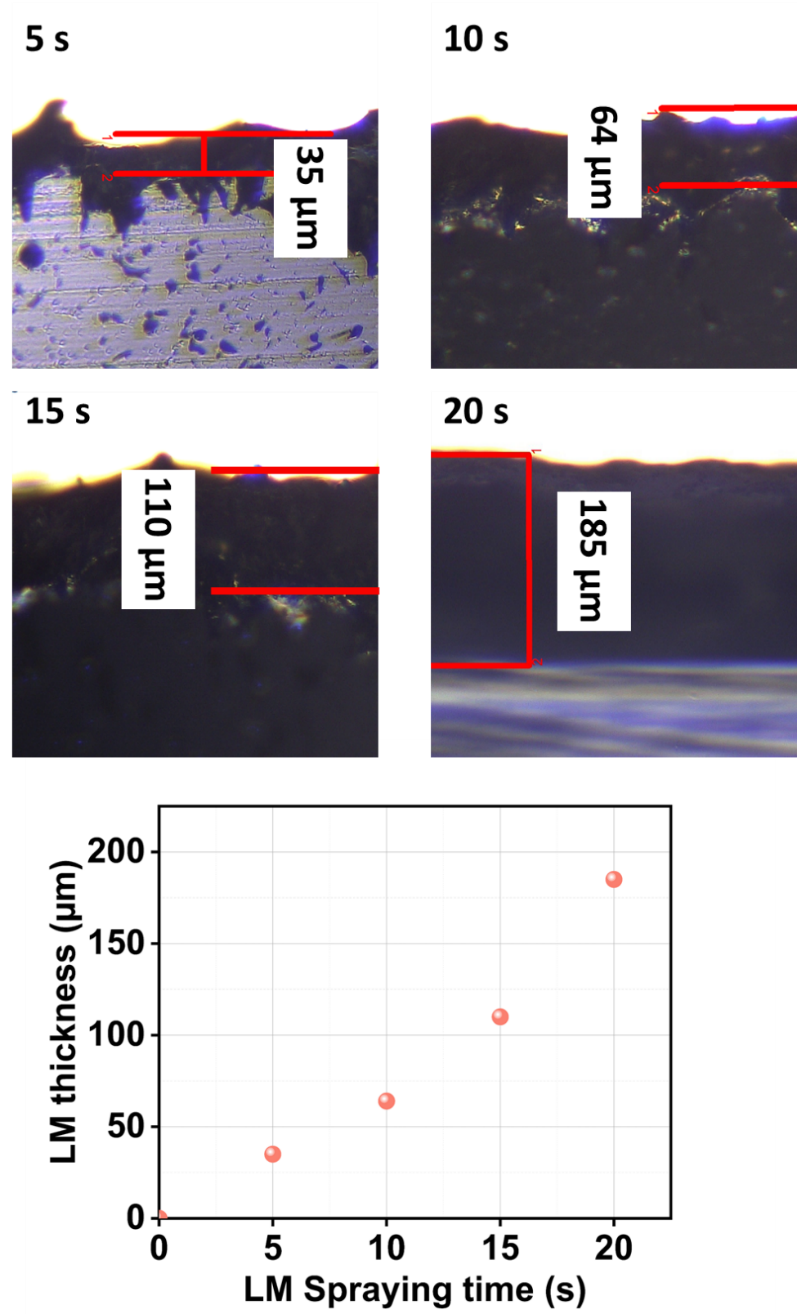


**Fig. S4** Optical microscope images of LM layers formed on silicone elastomer substrates by spray coating for 5, 10, 15, and 20 s, respectively (Top). The thicknesses of the liquid metal films as the function of spray coating duration time (Bottom)


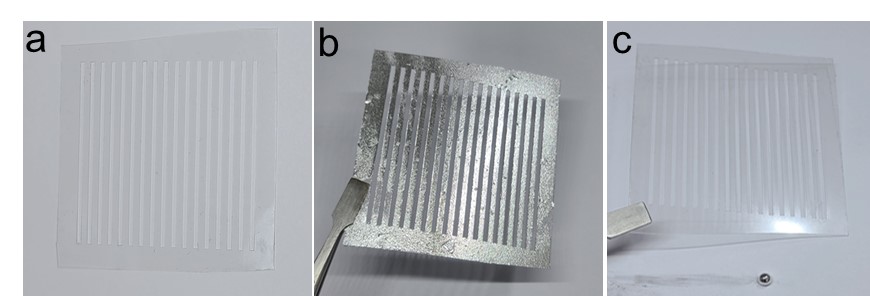


**Fig. S5** A stencil (**a**) before and (**b**) after spray coating liquid metal, and (**c**) after cleaning and recovering the deposited liquid metal on the stencil surface by swiping with .1M HCL solution saturated cotton tip and washing the stencil with detergent


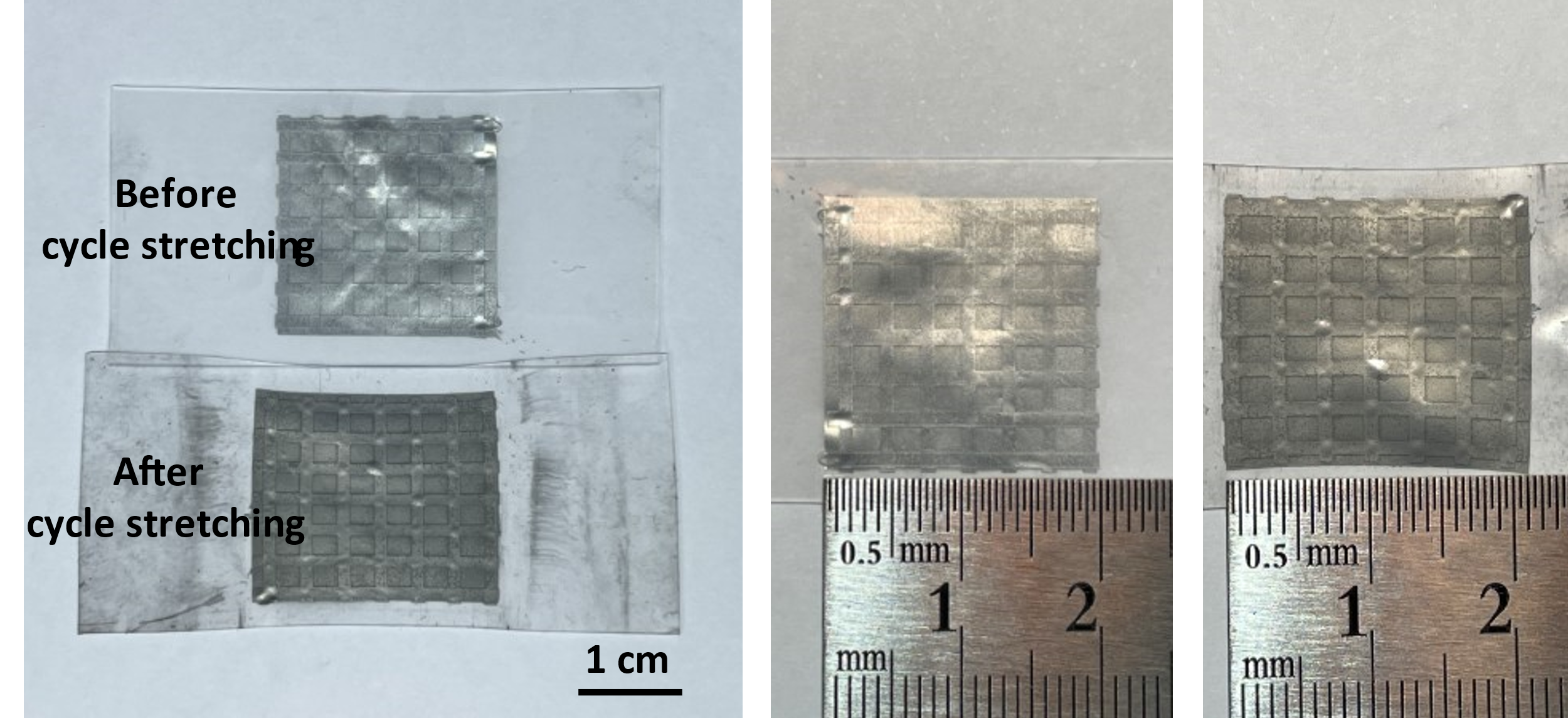


**Fig. S6** LMGD lengths measured before and after cycles of tensile strains


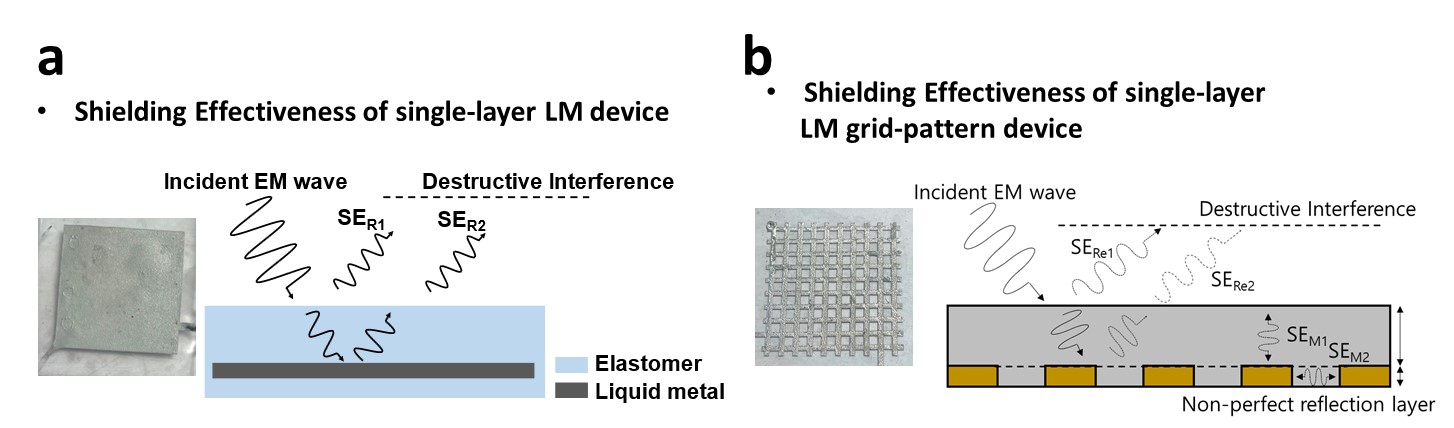


**Fig. S7** (**a, b**) Schematic showing the mechanism of the EMI shielding of (**a**) the single-layer LM device and (**b**) the single-layer LM grid-pattern device with the EMI shielding performance with frequencies ranging from 50 GHz to 110 GHz [S45]


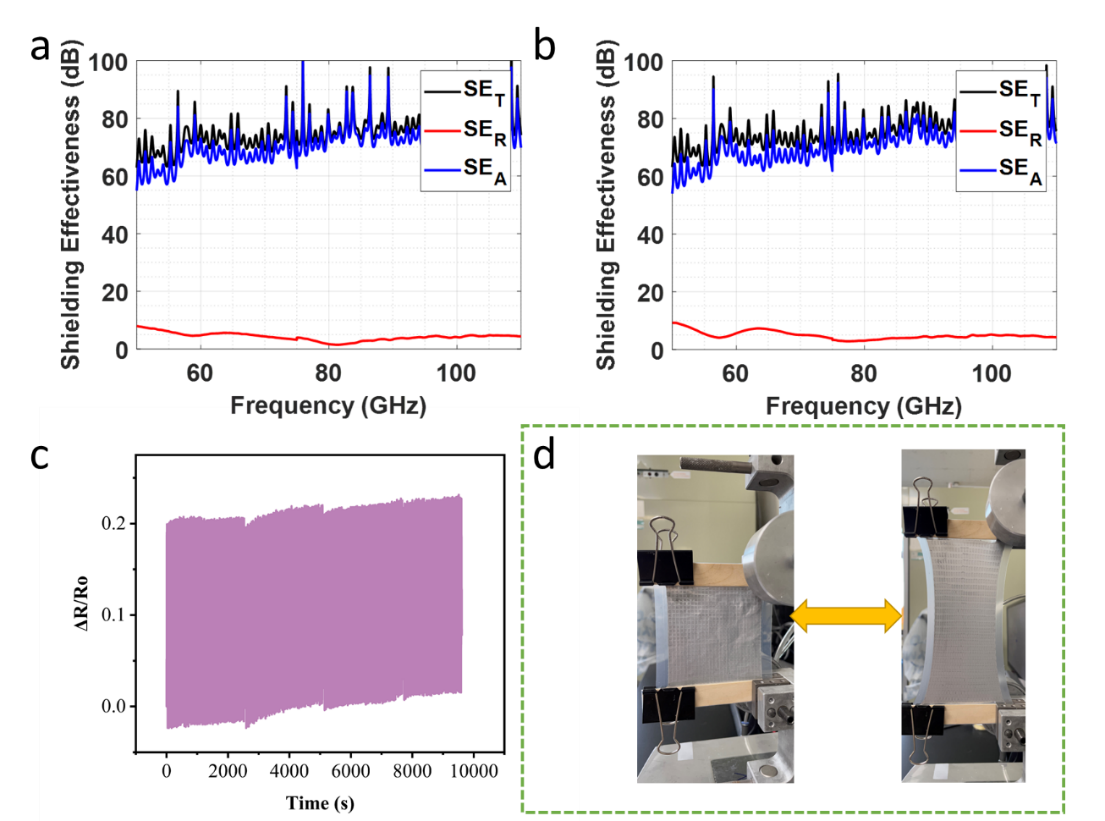


**Fig. S8** (**a, b**) The EMI SE of the LMGD with a 3 mm grid (**a**) before and (**b**) after applying 100 cycles of tensile strain. (**c**) Change in effective resistance of the LMGD during 250 cycles of loading and unloading tensile strain of 100%. (**d**) Photos showing the film upon loading and unloading tensile strain of 100%


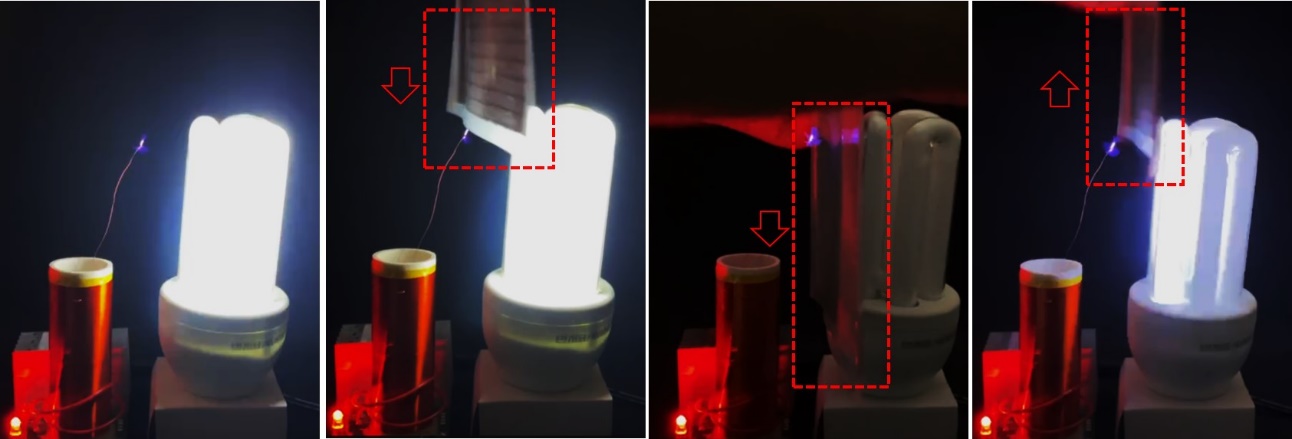


**Fig. S9** Digital photographs of a light powered by a Tesla coil turned off when interfered with LMGD


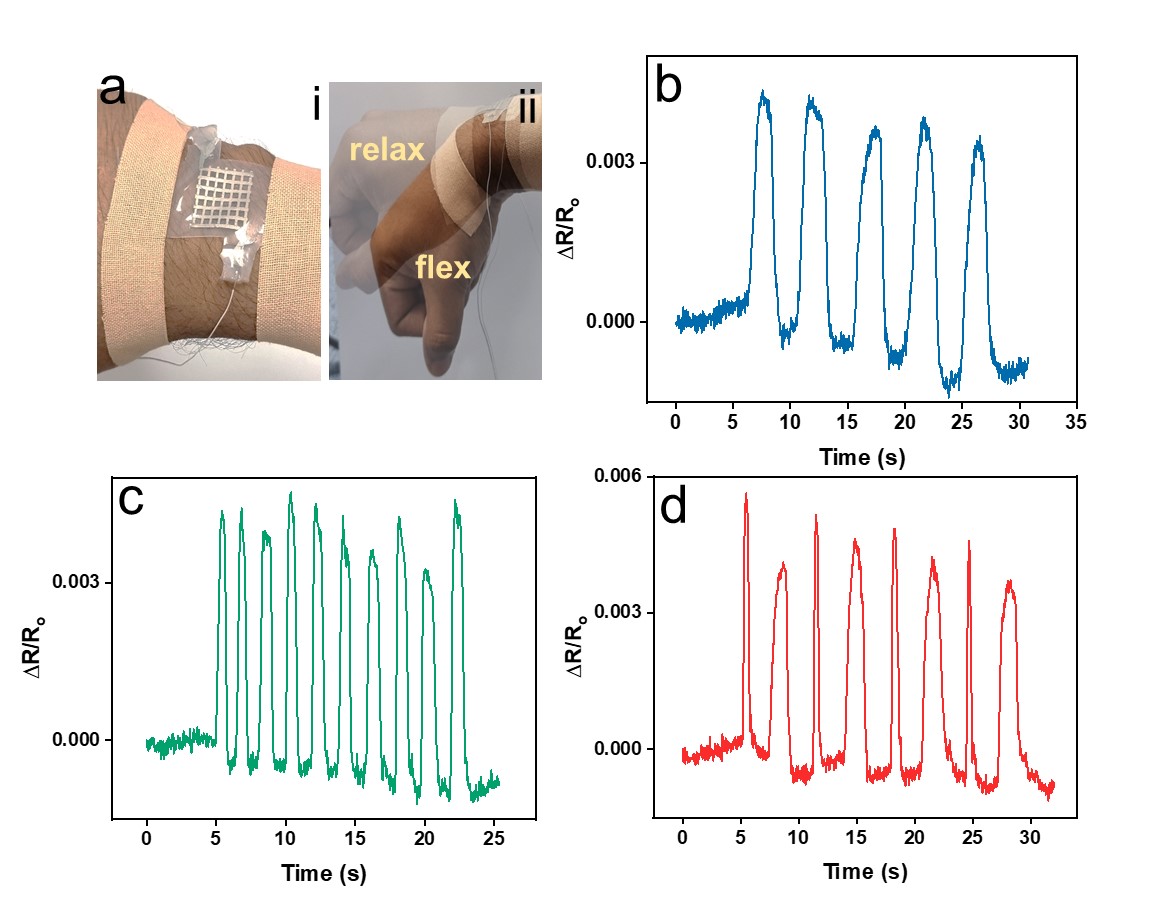


**Fig. S10** (**a**) The liquid metal grid patterned film being used as a biomechanical sensor to detect flexion and relaxation of the wrist while attached. (**b-d**) Resistance change profiles obtained during (**b**) slow, (**c**) fast and (**d**) periodic fast-slow flexing-relaxing movements

**Supplementary References**

1. J.G. Park, J. Louis, Q. Cheng, J. Bao, J. Smithyman et al., Electromagnetic interference shielding properties of carbon nanotube buckypaper composites. Nanotechnology **20**, 415702 (2009). <https://doi.org/10.1088/0957-4484/20/41/415702>
2. W.-L. Song, M.-S. Cao, M.-M. Lu, S. Bi, C.-Y. Wang et al., Flexible graphene/polymer composite films in sandwich structures for effective electromagnetic interference shielding. Carbon **66**, 67–76 (2014). <https://doi.org/10.1016/j.carbon.2013.08.043>
3. M.H. Al-Saleh, U. Sundararaj, Electromagnetic interference shielding mechanisms of CNT/polymer composites. Carbon **47**, 1738–1746 (2009). <https://doi.org/10.1016/j.carbon.2009.02.030>
4. R.B. Schulz, V.C. Plantz, D.R. Brush, Shielding theory and practice. IEEE Trans. Electromagn. Compat. **30**, 187–201 (1988). <https://doi.org/10.1109/15.3297>
5. M. Zhang, P. Zhang, Q. Wang, L. Li, S. Dong et al., Stretchable liquid metal electromagnetic interference shielding coating materials with superior effectiveness. J. Mater. Chem. C **7**, 10331–10337 (2019). <https://doi.org/10.1039/C9TC02887K>
6. Z. Wang, J. Ren, R. Liu, X. Sun, D. Huang et al., Three dimensional core-shell structured liquid metal/elastomer composite via coaxial direct ink writing for electromagnetic interference shielding. Compos. Part A Appl. Sci. Manuf. **136**, 105957 (2020). <https://doi.org/10.1016/j.compositesa.2020.105957>
7. M. Bayat, H. Yang, F.K. Ko, D. Michelson, A. Mei, Electromagnetic interference shielding effectiveness of hybrid multifunctional Fe_3_O_4_/carbon nanofiber composite. Polymer **55**, 936–943 (2014). <https://doi.org/10.1016/j.polymer.2013.12.042>
8. M. Crespo, N. Méndez, M. González, J. Baselga, J. Pozuelo, Synergistic effect of magnetite nanoparticles and carbon nanofibres in electromagnetic absorbing composites. Carbon **74**, 63–72 (2014). <https://doi.org/10.1016/j.carbon.2014.02.082>
9. A. Ameli, P.U. Jung, C.B. Park, Electrical properties and electromagnetic interference shielding effectiveness of polypropylene/carbon fiber composite foams. Carbon **60**, 379–391 (2013). <https://doi.org/10.1016/j.carbon.2013.04.050>
10. M.H. Al-Saleh, W.H. Saadeh, U. Sundararaj EMI shielding effectiveness of carbon based nanostructured polymeric materials: a comparative study. Carbon **60**, 146–156 (2013). <https://doi.org/10.1016/j.carbon.2013.04.008>
11. T. Kuang, L. Chang, F. Chen, Y. Sheng, D. Fu et al., Facile preparation of lightweight high-strength biodegradable polymer/multi-walled carbon nanotubes nanocomposite foams for electromagnetic interference shielding. Carbon **105**, 305–313 (2016). <https://doi.org/10.1016/j.carbon.2016.04.052>
12. M. Arjmand, M. Mahmoodi, G.A. Gelves, S. Park, U. Sundararaj, Electrical and electromagnetic interference shielding properties of flow-induced oriented carbon nanotubes in polycarbonate. Carbon **49**, 3430–3440 (2011). <https://doi.org/10.1016/j.carbon.2011.04.039>
13. Z. Zeng, H. Jin, M. Chen, W. Li, L. Zhou et al., Lightweight and anisotropic porous MWCNT/WPU composites for ultrahigh performance electromagnetic interference shielding. Adv. Funct. Mater. **26**, 303–310 (2016). <https://doi.org/10.1002/adfm.201503579>
14. N. Li, Y. Huang, F. Du, X. He, X. Lin et al., Electromagnetic interference (EMI) shielding of single-walled carbon nanotube epoxy composites. Nano Lett. **6**, 1141–1145 (2006). <https://doi.org/10.1021/nl0602589>
15. Y. Chen, H.-B. Zhang, Y. Yang, M. Wang, A. Cao et al., High-performance epoxy nanocomposites reinforced with three-dimensional carbon nanotube sponge for electromagnetic interference shielding. Adv. Funct. Mater. **26**, 447–455 (2016). <https://doi.org/10.1002/adfm.201503782>
16. Z. Zeng, M. Chen, H. Jin, W. Li, X. Xue et al., Thin and flexible multi-walled carbon nanotube/waterborne polyurethane composites with high-performance electromagnetic interference shielding. Carbon **96**, 768–777 (2016). <https://doi.org/10.1016/j.carbon.2015.10.004>
17. S. Kuester, C. Merlini, G.M.O. Barra, J.C. Ferreira, A. Lucas et al., Processing and characterization of conductive composites based on poly(styrene-b-ethylene-ran-butylene-b-styrene) (SEBS) and carbon additives: a comparative study of expanded graphite and carbon black. Compos. Part B Eng. **84**, 236–247 (2016). <https://doi.org/10.1016/j.compositesb.2015.09.001>
18. X. Jiang, D.-X. Yan, Y. Bao, H. Pang, X. Ji et al., Facile, green and affordable strategy for structuring natural graphite/polymer composite with efficient electromagnetic interference shielding. RSC Adv. **5**, 22587–22592 (2015). <https://doi.org/10.1039/c4ra11332b>
19. V.K. Sachdev, K. Patel, S. Bhattacharya, R.P. Tandon Electromagnetic interference shielding of graphite/acrylonitrile butadiene styrene composites. J. Appl. Polym. Sci. **120**, 1100–1105 (2011). <https://doi.org/10.1002/app.33248>
20. R. Sun, H.-B. Zhang, J. Liu, X. Xie, R. Yang et al., Highly conductive transition metal carbide/carbonitride(MXene)@polystyrene nanocomposites fabricated by electrostatic assembly for highly efficient electromagnetic interference shielding. Adv. Funct. Mater. **27**, 1702807 (2017). <https://doi.org/10.1002/adfm.201702807>
21. F. Shahzad, M. Alhabeb, C.B. Hatter, B. Anasori, S. Man Hong et al., Electromagnetic interference shielding with 2D transition metal carbides (MXenes). Science **353**, 1137–1140 (2016). <https://doi.org/10.1126/science.aag2421>
22. J. Liu, H.-B. Zhang, R. Sun, Y. Liu, Z. Liu et al., Hydrophobic, flexible, and lightweight MXene foams for high-performance electromagnetic-interference shielding. Adv. Mater. **29**, 1702367 (2017). <https://doi.org/10.1002/adma.201702367>
23. A. Iqbal, F. Shahzad, K. Hantanasirisakul, M.K. Kim, J. Kwon et al., Anomalous absorption of electromagnetic waves by 2D transition metal carbonitride Ti_3_CNT*_x_* (MXene). Science **369**, 446–450 (2020). <https://doi.org/10.1126/science.aba7977>
24. X. Huang, B. Dai, Y. Ren, J. Xu, P. Zhu, Preparation and study of electromagnetic interference shielding materials comprised of ni-co coated on web-like biocarbon nanofibers via electroless deposition. J. Nanomater. **2015**, 2 (2015). <https://doi.org/10.1155/2015/320306>
25. J. Li, S. Qi, M. Zhang, Z. Wang, Thermal conductivity and electromagnetic shielding effectiveness of composites based on Ag-plating carbon fiber and epoxy. J. Appl. Polym. Sci. **132**, e42306 (2015). <https://doi.org/10.1002/app.42306>
26. M.H. Al-Saleh, G.A. Gelves, U. Sundararaj, Copper nanowire/polystyrene nanocomposites: Lower percolation threshold and higher EMI shielding. Compos. Part A Appl. Sci. Manuf. **42**, 92–97 (2011). <https://doi.org/10.1016/j.compositesa.2010.10.003>
27. F. Fang, Y.-Q. Li, H.-M. Xiao, N. Hu, S.-Y. Fu, Layer-structured silver nanowire/polyaniline composite film as a high performance X-band EMI shielding material. J. Mater. Chem. C **4**, 4193–4203 (2016). <https://doi.org/10.1039/C5TC04406E>
28. A.A. Al-Ghamdi, F. El-Tantawy, New electromagnetic wave shielding effectiveness at microwave frequency of polyvinyl chloride reinforced graphite/copper nanoparticles. Compos. Part A Appl. Sci. Manuf. **41**, 1693–1701 (2010). <https://doi.org/10.1016/j.compositesa.2010.08.006>
29. J. Ling, W. Zhai, W. Feng, B. Shen, J. Zhang et al., Facile preparation of lightweight microcellular polyetherimide/graphene composite foams for electromagnetic interference shielding. ACS Appl. Mater. Interfaces **5**, 2677–2684 (2013). <https://doi.org/10.1021/am303289m>
30. D.-X. Yan, P.-G. Ren, H. Pang, Q. Fu, M.-B. Yang et al., Efficient electromagnetic interference shielding of lightweight graphene/polystyrene composite. J. Mater. Chem. **22**, 18772–18774 (2012). <https://doi.org/10.1039/C2JM32692B>
31. S.-T. Hsiao, C.-C M. Ma, H.-W. Tien, W.-H. Liao, Y.-S. Wang et al., Using a non-covalent modification to prepare a high electromagnetic interference shielding performance graphene nanosheet/water-borne polyurethane composite. Carbon **60**, 57–66 (2013). <https://doi.org/10.1016/j.carbon.2013.03.056>
32. Yan D.-X., Pang H., Li B., R. Vajtai, Xu L. et al., Structured reduced graphene oxide/polymer composites for ultra-efficient electromagnetic interference shielding. Adv. Funct. Mater. **25**, 559–566 (2015). <https://doi.org/10.1002/adfm.201403809>
33. A. Chaudhary, S. Kumari, R. Kumar, S. Teotia, B.P. Singh, Lightweight and easily foldable MCMB-MWCNTs composite paper with exceptional electromagnetic interference shielding. ACS Appl. Mater. Interfaces **8**, 10600−10608 (2016). <https://doi.org/10.1021/acsami.5b12334>
34. Y.-Q. Li, Y.A. Samad, K. Polychronopoulou, K. Liao, Lightweight and highly conductive aerogel-like carbon from sugarcane with superior mechanical and EMI shielding properties. ACS Sustainable Chem. Eng. **3**, 1419–1427 (2015). <https://doi.org/10.1021/acssuschemeng.5b00340>
35. B.-W. Li, Y. Shen, Z.-X. Yue, C.-W. Nan Enhanced microwave absorption in nickel/hexagonal-ferrite/polymer composites. Appl. Phys. Lett. **89**, 132504 (2006). <https://doi.org/10.1063/1.2357565>
36. K. Singh, A. Ohlan, P. Saini, S.K. Dhawan Poly (3, 4-ethylenedioxythiophene) *γ*-Fe_2_O_3_ polymer composite–super paramagnetic behavior and variable range hopping 1D conduction mechanism–synthesis and characterization. Polym. Adv. Technol. **19**, 229–236 (2008). <https://doi.org/10.1002/pat.1003>
37. S. Lin, S. Ju, G. Shi, J. Zhang, Y. He et al., Ultrathin nitrogen-doping graphene films for flexible and stretchable EMI shielding materials. J. Mater. Sci. **54**, 7165–7179 (2019). <https://doi.org/10.1007/s10853-019-03372-4>
38. D. Feng, D. Xu, Q. Wang, P. Liu Highly stretchable electromagnetic interference (EMI) shielding segregated polyurethane/carbon nanotube composites fabricated by microwave selective sintering. J. Mater. Chem. C **7**, 7938–7946 (2019). <https://doi.org/10.1039/C9TC02311A>
39. Y.-J. Wan, P.-L. Zhu, S.-H. Yu, R. Sun, C.-P. Wong et al., Ultralight, super-elastic and volume-preserving cellulose fiber/graphene aerogel for high-performance electromagnetic interference shielding. Carbon **115**, 629–639 (2017). <https://doi.org/10.1016/j.carbon.2017.01.054>
40. J. Jung, H. Lee, I. Ha, H. Cho, K.K. Kim et al., Highly stretchable and transparent electromagnetic interference shielding film based on silver nanowire percolation network for wearable electronics applications. ACS Appl. Mater. Interfaces **9**, 44609–44616 (2017). <https://doi.org/10.1021/acsami.7b14626>
41. P. Li, D. Du, L. Guo, Y. Guo, J. Ouyang Stretchable and conductive polymer films for high-performance electromagnetic interference shielding. J. Mater. Chem. C **4**, 6525–6532 (2016). <https://doi.org/10.1039/c6tc01619g>
42. B. Yao, W. Hong, T. Chen, Z. Han, X. Xu et al., Highly stretchable polymer composite with strain-enhanced electromagnetic interference shielding effectiveness. Adv. Mater. **32**, e1907499 (2020). <https://doi.org/10.1002/adma.201907499>
43. X. Jiang, J. Zhou, X. Zhong, Z. Hu, R. Hu et al., Stretchable PEDOT: PSS/Li-TFSI/XSB composite films for electromagnetic interference shielding. ACS Appl. Mater. Interfaces **15**, 8521–8529 (2023). <https://doi.org/10.1021/acsami.2c21604>
44. Q. Li, Y. Sun, B. Zhou, G. Han, Y. Feng et al., Flexible, stretchable, and transparent MXene nanosheet/thermoplastic polyurethane films for multifunctional heating and electromagnetic interference shielding. ACS Appl. Nano Mater. **6**, 3395–3404 (2023). <https://doi.org/10.1021/acsanm.2c05169>
45. S.H. Ryu, Y.K. Han, S.J. Kwon, T. Kim, B.M. Jung et al., Absorption-dominant, low reflection EMI shielding materials with integrated metal mesh/TPU/CIP composite. Chem. Eng. J. **428**, 131167 (2022). <https://doi.org/10.1016/j.cej.2021.131167>
